# Diversity of the southern Africa *Lacustricola* Myers, 1924 and redescription of *Lacustricola johnstoni* (Günther, 1894) and *Lacustricola myaposae* (Boulenger, 1908) (Cyprinodontiformes, Procatopodidae)

**DOI:** 10.3897/zookeys.923.48420

**Published:** 2020-04-01

**Authors:** Pedro H.N. Bragança, Ryan M. van Zeeventer, Roger Bills, Denis Tweddle, Albert Chakona

**Affiliations:** 1 South African Institute for Aquatic Biodiversity, Private Bag 1015, Grahamstown, 6140, South Africa South African Institute for Aquatic Biodiversity Grahamstown South Africa; 2 Department of Ichthyology and Fisheries Science, Rhodes University, Grahamstown, South Africa Rhodes University Grahamstown South Africa

**Keywords:** African lampeyes, diversity, DNA barcoding, fish, freshwater, taxonomy, topminnows

## Abstract

Through the analysis of a comprehensive database of COI sequences, with the sequencing of 48 specimens, a first insight into the genetic diversity, distribution and relationships between the southern Africa “*Lacustricola*” species is presented. Species from “*Lacustricola*” occur mainly in freshwater systems within the arid savanna, and are considered to be widely distributed in southern Africa, but most of them are data deficient taxa. Two species are redescribed, “*Lacustricola*” *johnstoni* (Günther, 1894) and “*Lacustricola*” *myaposae* (Boulenger, 1908), based on specimens collected at their respective type localities. Detailed osteological and life colouration information is presented for the first time. “*Lacustricola*” *johnstoni* was described from the Upper Shire River in Mangochi, Lake Malawi but is herein considered as widespread in the Okavango, Zambezi, southern Africa east coastal drainages and the Bangweulu in the Congo System. A sympatric similar species occurring in the Okavango is also identified. “*Lacustricola*” *myaposae* (Boulenger, 1908), was described from the Nseleni River in KwaZulu-Natal Province, South Africa and is herein considered to be endemic to the small coastal river drainages within this region. Lectotypes for both “L.” *johnstoni* and “L.” *myaposae* are designated. A new species from the Lualaba River in the Congo System, sister to “L.” *macrurus* is identified, and the deep bodied “L.” *jubbi* is considered sister taxon to a clade including “L.” *johnstoni* and “L.” *myaposae*.

## Introduction

The Procatopodidae comprises approximately 100 small oviparous killifishes distributed across the major African freshwater systems (Ghedotti 2000; Bragança and Costa 2019). Fishes of this family are popularly known as African lampeyes due to the presence of a characteristic iridescent reflective colouration in the dorsal region of the eye. Historically, a close relationship between the procatopodids, the Amazon miniature oviparous killifish species of *Fluviphylax* Whitley, 1965 and the American live-bearing poeciliids had been suggested based on morphological phylogenies (Parenti 1981; Costa 1996; Ghedotti 2000). Subsequent investigation of molecular data provided better insights into the relationships among the main clades of Cyprinodontiformes (Pollux et al. 2014; Pohl et al. 2015; Helmstetter et al. 2016; Reznick et al. 2017; Bragança et al. 2018; Bragança and Costa 2018). More recently, Bragança et al. (2018) provided a comprehensive molecular phylogeny of the Cyprinodontiformes based on sequences of one mitochondrial and five nuclear genes. Results from this study refuted monophyly of the family Poeciliidae, and the African lampeyes were assigned to the family Procatopodidae which is considered to be sister group of the Old World Aphaniidae and Valenciidae (Bragança et al. 2018). Findings from these aforementioned studies stimulated further interest in establishing the relationships among the African procatopodids.

Bragança and Costa (2019) published a more inclusive molecular time calibrated phylogenetic analysis directed to assess the internal relationships among the little known African lampeye genera, revealing the timing and diversification patterns among procatopodids and evidencing the paraphyly of some of its proposed genera. According to Bragança and Costa (2019), Procatopodidae split from Aphaniidae and Valenciidae as a consequence of the trans-Saharan sea retreat during the Late Eocene-Early Oligocene transition, and most clades diversified during the moist-wet climate stability period from the Late Oligocene/Early Miocene until the Middle Miocene. However, the extreme aridification and climatic instability seen in the Late Miocene and in the Pliocene-Pleistocene respectively, probably promoted diversification of one particular African lampeye clade in savannahs and arid environments. Within this clade there are the widely distributed “*Poropanchax*” normani (Ahl, 1928), and species belonging to *Micropanchax* Myers, 1924, *Poropanchax* Clausen, 1967, *Rhexipanchax* Huber, 1999 and the southern Africa “*Lacustricola*” Myers, 1924 species (Bragança and Costa 2019).

The genus *Lacustricola* was found to be polyphyletic, i.e. with two distinct and non-related species groups. The first group comprised all eastern Africa species including the type species of the genus, *L. pumilus* (Boulenger, 1906); and the second group comprises all southern Africa species (Bragança and Costa 2019). The southern Africa *Lacustricola* species were considered to be sister to *Micropanchax* and both these genera contain species that are currently considered to have broad distribution ranges across the African savannah associated water systems (Bragança and Costa 2019). Ongoing critical evaluation integrating molecular, morphological and osteological data support the proposed split of *Lacustricola* (Bragança unpublished data). This work will result in the establishment of new generic names, but in the present study, *Lacustricola* refers to species belonging to the eastern Africa clade and “*Lacustricola*” to species from the southern Africa clade, following Bragança and Costa (2019).

The southern “*Lacustricola*” clade is much more diverse than the eastern clade. Species in the southern clade have broad distribution ranges across most river systems in southern Africa, from the Nseleni in the south through the Okavango, Cunene, Kwanza, Zambezi, coastal river drainages in Mozambique and tributaries of the Congo (Wildekamp et al. 1986; Skelton 2001). The deep bodied species previously placed in the genus *Hypsopanchax* Myers, 1924, such as *H. jubbi* Poll & Lambert, 1965, *H. jobaerti* Poll & Lamberti, 1965 and *H. stiassnyae* Van der Zee, Sonnenberg & Mbimbi Mayi Munene, 2015 were recently found to belong to the southern *Lacustricola* clade (Bragança and Costa 2019; Bragança unpublished data). There are three main subgroups within the southern clade. These are the “L.” katangae (Boulenger, 1912) group which is defined by a zigzag black mark along the lower portion of the flank, the “L.” hutereaui (Boulenger, 1913) group which is defined by the presence of barred dorsal, anal and caudal-fins and a conspicuous reticulate pattern on scale margins, and the “L.” *johnstoni* (Günther,1894) group which is defined by a slender body and lack of the aforementioned characters. All three subgroups have broadly similar distribution patterns, with some species occurring sympatrically.

The present study builds and expands on previous efforts by incorporating a comprehensive database of mitochondrial COI sequences (“DNA-barcodes”) to examine the diversity and map the distribution of species and lineages in the southern “*Lacustricola*” clade. However, because of considerable sampling gaps there is lack of data for some topotypes of currently recognised species and their synonyms; therefore, the purpose of the present study is to provide a first snapshot of the diversity within the southern “*Lacustricola*” clade and provide a roadmap for future taxonomic revision of this group. The paper also provides updated descriptions and diagnoses for “L.” *johnstoni* (Günther, 1894) and “L.” *myaposae* (Boulenger, 1908) based on data from comprehensive conspecific topotypic specimens of these species, respectively, in Mangochi, Lake Malawi, Malawi and in the Nseleni River, in KwaZulu Natal, South Africa, as well as examination of the species syntypes. We have also selected and designated the following specimens as lectotypes for “L.” *johnstoni* (BMNH 1893.11.15.95) and “L.” *myaposae* (BMNH 1907.4.17.88), to contribute to the ongoing effort in studying and describing the southern Africa “*Lacustricola*” diversity.

## Materials and methods

### Specimens examined, preservation, and fixation

The present study included specimens and tissue samples that were collected from historical surveys and recent expeditions in southern Africa and were deposited into the National Collection Facility at the NRF-South African Institute for Aquatic Biodiversity (**NRF-SAIAB**), the Federal University of Rio de Janeiro (**UFRJ**) in Rio de Janeiro, Brazil and the Royal Museum for Central Africa (**RMCA**) in Tervuren, Belgium. “*Lacustricola*” *johnstoni* and “L.” *myaposae* syntypes from the Natural History Museum (**BMNH**), London, UK were examined from photographs, and lectotypes were designated. Fishes were sampled using various gears including electrofishing, seine nets, traps/fyke nets and dip nets. Captured fishes were anaesthetised with clove oil, digitally photographed and a small piece of muscle tissue was dissected from the right side of each specimen and preserved in 95% ethanol in the field for genetic analysis. Tissue samples were stored at -80 °C at the NRF-SAIAB, Grahamstown. Voucher specimens were fixed in 10% formalin in the field and were then transferred through 10% and 50% to 70% ethanol for long-term storage upon returning from the field. Specimens examined for the redescription of “L.” *johnstoni* and “L.” *myaposae* are listed in the taxonomic accounts section. A list of samples included in the molecular analysis with their respective localities and GenBank accession numbers are presented in Suppl. material 1.

### Morphological study and osteological preparations

Meristics and morphometric data of “L.” *johnstoni* and “L.” *myaposae* were taken from specimens fixed in formalin and transferred to 70% ethanol (material listed in the taxonomy accounts section). Body measurements are presented as proportions of standard length (SL) and head measurements are expressed as proportions of head length (HL). Measurements were obtained using digital callipers under a dissecting microscope following Costa (1988). Osteological studies were made on cleared and stained specimens prepared according to Taylor and Dyke (1985), and nomenclature for bone structures followed Costa (2006). Most osteological illustrations were made on the left side, unless these were damaged. Nomenclature for frontal squamation follows Hoedeman (1956) and that for head sensory canals follows Gosline (1949), except for the posterior section of supraorbital canal, here called the posterior infraorbital canal.

### Taxon sampling

Mitochondrial COI sequences of 48 specimens representing most of the southern Africa “*Lacustricola*” species were included in this study. In addition to the sequences produced in this study, other sequences were selected from GenBank (Suppl. material 1). We generated COI sequences for samples of conspecific specimens (topotypes) collected close to the type localities of “L.” *johnstoni*, “L.” *myaposae* and “L.” katangae and these were designated as topogenetypes following Chakrabarty (2010). Information on the locality details of samples used and GenBank accession numbers are presented in Suppl. material 1. The procatopodid species “*Poropanchax*” normani which is known as sister to all other genera and species within the savannah and arid environment clade (Bragança and Costa 2019) was selected as outgroup. Species belonging to the southern Africa *Lacustricola* clade will be referred to as belonging to “*Lacustricola*”, following Bragança and Costa (2019), indicating that species from the southern Africa clade are not related to *L. pumilus*, the genus type species.

### DNA extraction, PCR, and sequencing

DNA was extracted from preserved tissues using the salting out method (Sunnucks et al. 1996) and by using the GeneJet Genomic DNA Purification kit (Thermo Fisher Scientific) and the NucleoSpin Tissue kit (Machery-Nagel Gmbh & Co. KG) following the manufacturer’s standard protocol for animal tissue isolation. A fragment of the mitochondrial cytochrome oxidase subunit I (COI) gene was amplified by polymerase chain reaction (PCR) using the general universal DNA barcoding primer set: LCO1490 and HCO 2198 (Folmer et al. 1994). PCRs were performed with a Veriti 96 well thermal cycler (Applied Biosystems) and each reaction mixture (25 μL) contained 100–200 ng template DNA, 14.4 μL of water, 2.5 μL deoxynucleotide triphosphate (dNTP) (10 mM), 2.5 mM MgCl_2_, 2.5 μL PCR buffer (10X), 0.5 μL of each primer (20 pmol) and 0.1 μL *Taq* DNA polymerase (Southern Cross Biotechnology, Cape Town). The PCR amplification profile was 95 °C for 5 min, followed by 35 cycles of 95 °C for 1 min, 43–47 °C for 1min and 72 °C for 2 min, and then final extension at 72 °C for 7 min. PCR products were purified with Exosap (Applied Biosystems), cycle-sequenced using BigDye Cycle Sequencing Kit (Applied Biosystems, Foster City, CA, USA) and sequenced at the NRF-SAIAB using an ABI 3730xl DNA Analyzer (Applied Biosystems) or in Macrogen, South Korea.

### Alignment, evolution model, and phylogenetic analysis

Sequences were cleaned, aligned and trimmed to equal lengths (676 bp) using the program MEGA 7.0 (Kumar et al. 2016). The sequence evolution model HKY+I+G was selected using the corrected Akaike’s Information Criteria (AICc) as implemented in JMODELTEST 2 (Darriba et al. 2012). Phylogenetic analyses were conducted through Bayesian inference (BI), using the program MRBAYES v3.2.5 (Ronquist et al. 2012) and Maximum Likelihood (ML), using the program GARLI 2.0 (Zwickl 2006). When performing MRBAYES v3.2.5, BI was conducted using two Markov chain Monte Carlo (MCMC) runs of four chains each for 3 million generations and a sampling frequency of 1000. The quality of the MCMC chains, stationarity and the respective ESS values of analysis parameters were checked in Tracer 1.6, and the analysis was finished when parameters were above 200. A 25% burn-in was removed in MRBAYES v3.2.5. A Maximum Likelihood stepwise-addition starting tree was performed in GARLI 2.0, with 100 attachment branches for each taxon and ten independent search replicates. The support values of the ML analysis were calculated by 1000 bootstrap replications (Felsenstein 1985).

## Results

### Molecular phylogenetic analyses

**Figure 1.**
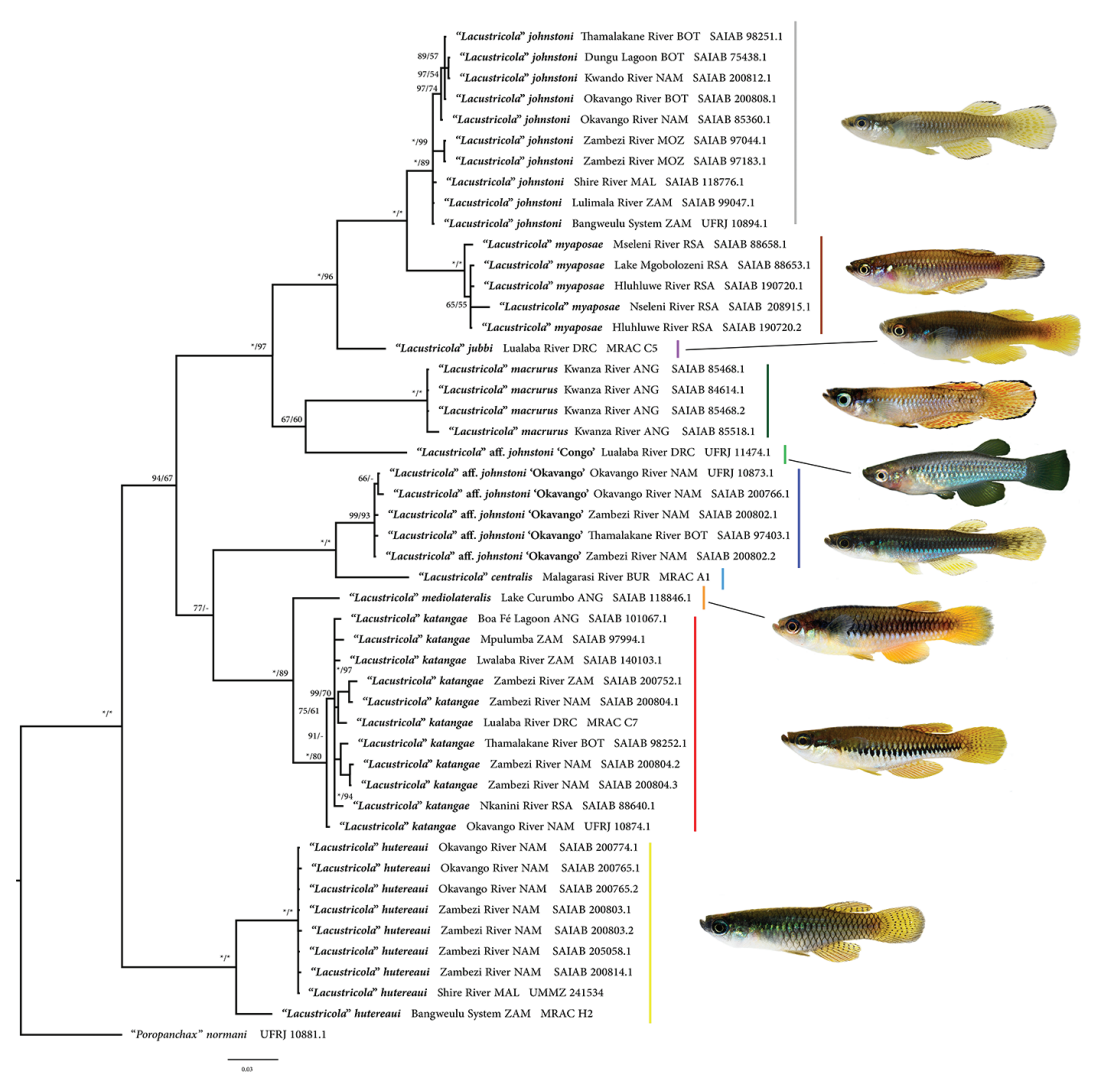
Phylogenetic relationships between southern Africa “*Lacustricola*” haplotypes, based on COI mitochondrial DNA sequences. Numbers left to the bar indicate posterior probability values and on the right are bootstrap support values from the maximum likelihood analysis. Asterisks indicate maximum values. Colours next to each species name correspond to the same colours as depicted in the distribution map (Figure 2). Abbreviations refer to the country where the specimens were collected: ANG = Angola, BOT = Botswana, BUR = Burundi, DRC = Democratic Republic of Congo, NAM = Namibia, MAL = Malawi, MOZ = Mozambique, RSA = Republic of South Africa, ZAM = Zambia.

**Figure 2.**
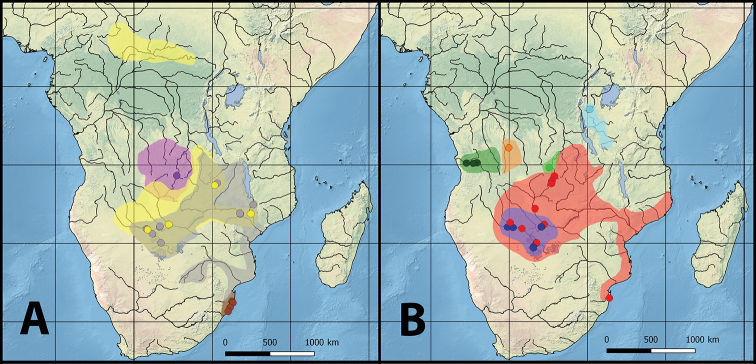
Southern Africa “*Lacustricola*” distribution maps. Spots correspond to the exact localities for the haplotypes included in this study, and the shaded area refers to the inferred distribution for each species. Map **A** yellow – “*Lacustricola*” hutereaui; grey – “L.” *johnstoni*; brown – “L.” *myaposae*; and purple – “L.” *jubbi.* Map **B** dark green – “L.” macrurus; orange – “L.” mediolateralis; red – “L.” katangae; light blue – “L.” centralis; blue – “L. ” aff. johnstoni ‘Okavango’; light green – “L. ” aff. johnstoni ‘Congo’.

ML and the BI analyses recovered trees with comparable topologies (Fig. 1). Haplotypes belonging to the ‘”*Lacustricola*” hutereaui group were recovered as sister to all the southern African “*Lacustricola*” haplotypes, being supported by maximum support values (Fig. 1). The “L.” hutereaui group contains two genetic lineages, one comprising haplotypes derived from specimens of the Zambezi and Okavango region and the other comprising a haplotype from the Lualaba River, a major tributary of the Congo River basin (Fig. 2). Haplotypes of “*Lacustricola*” katangae and “L.” *mediolateralis* (Poll, 1967) were recovered as sister groups. “*Lacustricola*” katangae haplotypes were found to be broadly distributed, occurring in the Congo, Upper Zambezi and Okavango river systems (Figs 1, 2).

Haplotypes including “L.” centralis (Seegers, 1996), from the Malagarasi River in eastern Africa, and an undescribed species which occurs in the Okavango drainage, herein named “Lacustricola ” aff. johnstoni “Okavango” were recovered as sister groups (Figs 1, 2). However, the relationship between haplotypes of “L.” centralis, “L. ” aff. johnstoni “Okavango”, “L.” katangae and “L.” mediolateralis had low support. The analyses recovered a well-supported clade containing “L.” *johnstoni* s.s., “L.” myaposae, “L.” *jubbi*, “L.” macrurus and an undescribed species, “L. ” aff. johnstoni “Congo” (Fig. 1). Haplotypes of “*Lacustricola*” *jubbi* were recovered as the basally diverging lineage which is sister to a clade containing two sister species, “L.” *myap*osae and “L.” *johnstoni* s.s.

“*Lacustricola*” *johnstoni* s. s. is widely distributed in southern Africa, with a range extending from the Lower Zambezi system (i.e., the Shire River/Lake Malawi system), through the Middle and Upper Zambezi to the Okavango system and the Bangweulu catchment in the Congo River system (Fig. 2). “*Lacustricola*” myaposae is endemic to the eastward draining river systems of the Maputaland region in South Africa and Mozambique (Fig. 2). “*Lacustricola*” *jubbi*, which was previously placed in the deep bodied *Hypsopanchax* genus occurs in the Upper Zambezi from where it was first described, and in the Lualaba River, Upper Congo River system, where it co-occurs with “L. ” aff. johnstoni “Congo” (Fig. 2). “*Lacustricola*” macrurus is endemic to the Kwanza River system and the adjacent Lake Sarmento at Marimba, Angola, that drains into the Congo River system (Fig. 2). Given the existence of several unidentified lineages within the southern “*Lacustricola*” clade, the present study aims to provide comprehensive redescriptions of “L.” *johnstoni* s. s. and “L.” myaposae as part of a long-term effort to revise the taxonomy of this genus.

### Taxonomic accounts

#### Lacustricola johnstoni

Taxon classificationAnimaliaCyprinodontiformesPoeciliidae

(Günther, 1894)

94E26DE3-0DE7-54CE-8A7B-CBBCE029BE57

Figures 3 , 4

Haplochilus johnstoni Günther, 1894: 627 [original description: Mangochi (former Fort Johnston), Malawi].

##### Material examined.

BMNH 1893.11.15.95, Lectotype; BMNH 1893.11.15.92-94,96-99, 7 Paralectotypes; Mangochi (former Fort Johnston), Malawi. Examined by photographs and x-rays – SAIAB 35820, 18 (5 C&S), 24.7–35.4 mm SL; Upper Shire River, Mangochi, Malawi, 14°26'60"S, 35°15'60"E; col: D. Tweddle & N. G. Willoughby; 19 Sep. 1971. – SAIAB 8311, 3, 31.3–32.5 mm SL; Shire River, Liwonde, Malawi; col: D. Tweddle & N. G. Willoughby; 20 Oct. 1975. – SAIAB 34384, 1, 29.1 mm SL; Shire River, Liwonde Barrage, Malawi, 15°04'S, 35°13'E; col: D. Tweddle & P. Skelton; 26 Oct. 1989. – SAIAB 34388, 1, 35.7 mm SL; Monkey Bay, Lake Malawi, Malawi, 14°04'S, 34°55'E; 17 Oct. 1989. – SAIAB 40800, 15 (4 C&S), 30.1–33.2 mm SL; Bridge over Dwambadzi River, Malawi, 12°14'S, 33°59'E; col: D. Tweddle & P. Skelton; 06 Sep. 1992. – SAIAB 11237, 7, 26.2–36.2 mm SL; Monkey Bay, Lake Malawi, Malawi, 14°3'00"S, 34°55'00"E; col: D.H. Eccles; 31 Oct. 1974. – SAIAB 11876, 8, 27.2–34.7 mm SL; Shire River, Liwonde Barrage, Malawi, 15°3'37"S, 35°13'7"E; col: D. Tweddle & T. Makinen; 27 May. 2011.

##### Diagnosis.

“*Lacustricola*” *johnstoni* is distinguished from all congeners from the “L.” katangae clade by the absence of a zigzag black mark along the flank (vs. presence); and from congeners belonging to the “L.” hutereaui clade by the absence of a barred dorsal, anal and caudal-fins and also by the absence of a conspicuous reticulate pattern on scale margins. It is further distinguished from all congeners except “L.” *myaposae* and “L.” *moeruensis* by the presence of orange dorsal, anal and caudal-fins in females (vs. hyaline); it is distinguished from “L.” *myaposae* by the presence of a bluish colouration in the posterior region of flank (vs. light purple colouration); a slender body profile, male body depth 20.6–24.4% of SL (vs. 26.0–30.9% of SL), female body depth 19.7–22.5% of SL (vs. 22.8–25.1% of SL); a shorter dorsal-fin base length in males 8.7–11.6% of SL (vs. 11.9–13.1% of SL) and in females 7.3–10.1% of SL (vs. 10.8–11.6% SL); a less deep head in males 61.9–67.0% of HL (vs. 70.7–79.8% of HL) and in females 59.4–63.5% of HL (vs. 66.6–69.6% of HL); and a hyaline pectoral-fin in males (vs. orange). Other morphometric characters presenting a slight overlap but useful in distinguishing “L.” *johnstoni* from “L.” *myaposae* are: a comparatively narrow caudal peduncle, depth of 12.6–14.5% of SL in males and 11.2–12.7% of SL in females (vs. 14.0–17.1% of SL in males and 12.9–13.7% of SL in females); a comparatively elongated caudal-fin, 30.1–33.8% of SL in males and 28.9–31.2% of SL in females (vs. 27.5–30.2% of SL in males and 25.3–28.5% of SL in females); and a comparatively deep, laterally compressed head, 57.1–63.2% of HL in males and 59.0–64.7% of HL in females (vs. 63.1–67.9% of HL in males and 65.3–68.1% of HL in females). “*Lacustricola*” *johnstoni* is distinguished from “L.” *moeruensis* by a comparatively slender body and a more backward positioned dorsal-fin, first proximal radial of dorsal-fin between neural spine of vertebrae 16 and 17 (vs. 13 and 14).

##### Description.

Morphometric data are presented in Table 1. Maximum recorded adult size 35.6 mm SL. Dorsal profile of body approximately straight to slightly convex from snout tip to dorsal-fin origin; convex along dorsal-fin base. Ventral profile convex from lower jaw to beginning of anal-fin base; slightly convex along the anal-fin base and nearly straight on caudal peduncle. Anterior portion of body laterally compressed, becoming more compressed behind anal-fin origin.

**Table 1.** Morphometric data of “*Lacustricola*” *johnstoni* and “L.” *myaposae*.

	“*Lacustricola*” *johnstoni*		“*Lacustricola*” *myaposae*	
	males (*N* = 12)	females (*N* = 10)	males (*N* = 7)	females (*N* = 4)
Standard length (mm)	29.0–34.6	27.7–35.6	31.3–38.1	30.2–39.0
**Percent of standard length**				
Body depth	20.6–24.4	19.7–22.5	26.0–30.9	22.8–25.1
Caudal peduncle depth	12.6–14.5	11.2–12.7	14.0–17.1	12.9–13.7
Pre-dorsal length	67.6–73.9	71.6–73.9	67.5–72.3	68.5–70.7
Pre-pelvic length	43.0–48.6	44.9–49.7	45.1–49.5	46.4–47.8
Length of dorsal-fin base	8.7–11.6	7.3–10.1	11.9–13.1	10.8–11.6
Length of anal-fin base	16.8–21.1	13.2–16.6	16.0–20.0	15.0–16.2
Caudal-fin length	30.1–33.8	28.9–31.2	27.5–30.2	25.3–28.5
Pectoral-fin length	18.6–22.1	18.8–20.8	18.7–22.4	17.5–19.5
Pelvic-fin length	15.5–22.8	13.2–14.6	14.0–18.0	11.9–12.9
Head length (mm)	6.9–8.4	6.2–7.9	8.2–9.5	7.2–9.8
**Percent of head length**				
Head depth	61.9–67.9	59.4–63.5	70.7–79.8	66.6–69.6
Head width	57.1–63.2	59.0–64.7	63.1–67.9	65.3–68.1
Snout length	21.0–23.9	19.2–23.4	20.7–22.8	22.2–24.4
Lower jaw length	9.5–12.3	8.7–11.0	9.8–14.3	8.9–11.2
Eye diameter	35.7–39.5	36.2–41.1	35.8–38.5	37.2–39.2

**Figure 3.**
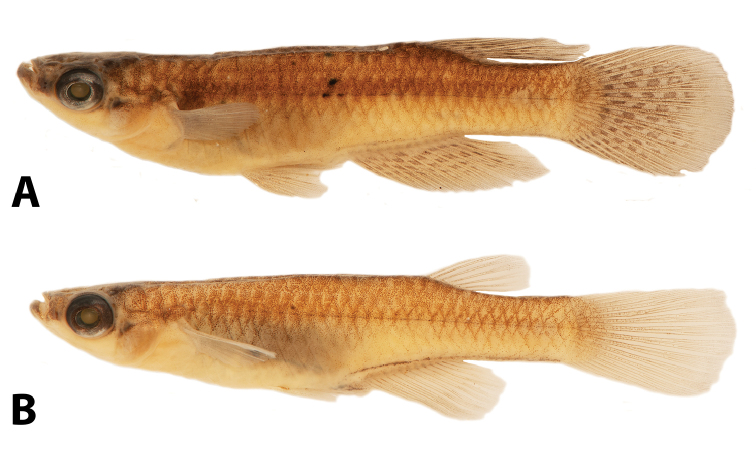
“*Lacustricola*” *johnstoni* preserved colouration: **A** male SAIAB 118776, 34.7 mm SL**B** female SAIAB 118776, 32.0 mm SL; from Shire River at Liwonde Barrage, Malawi.

**Figure 4.**
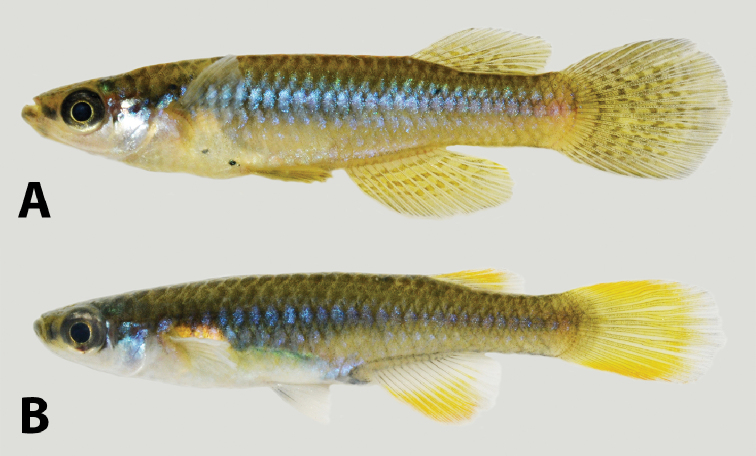
“*Lacustricola*” *johnstoni* colouration in life: **A** male SAIAB 118776, 31.0 mm SL**B** female SAIAB 118776, 26 mm SL; from Shire River at Liwonde Barrage, Malawi.

There is clear sexual dimorphism in fin shape and size (Figs 3, 4). In males, dorsal-fin is rounded and elongated, almost reaching the caudal-fin base; its origin in vertical between 7^th^ and 9^th^ anal-fin rays; anal-fin rounded and elongated, reaching middle of the caudal peduncle. Pelvic-fin length variable, reaching between urogenital papillae aperture and the base of third anal-fin ray. In females, dorsal and anal-fins are not elongated and do not extend posteriorly to caudal-fin base. Caudal-fin slender in both sexes. Pectoral-fin elliptical, in both males and females, its posterior margin reaching vertical just behind pelvic-fin base. In females, pelvic-fin shorter than in males, tip reaching region just before urogenital opening. In both males and females, dorsal-fin rays 7(17), 8(13) and 9(2); anal-fin rays 12(4), 13(12), 14(14) and 15(2); caudal-fin rays 19(2), 20(10), 21(17), 22(2) and 23(1); pectoral-fin rays 12(13), 13(18) and 14(1); pelvic-fin rays 6.

**Figure 5.**
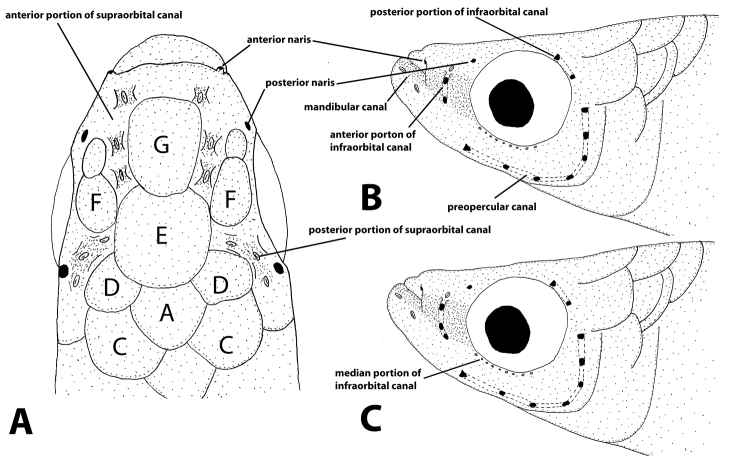
Cephalic pores and head squamation pattern: **A, B** head dorsal and lateral view of “L.” *johnstoni***C** head lateral view of “L.” *myaposae*.

Frontal squamation G-patterned (Fig. 5). Head neuromasts placed in shallow grooves. Cephalic lateral line system: anterior portion of supraorbital sensory canal open, with three neuromasts, anteriormost one anteriorly displaced from the other two; posterior portion open, with three exposed neuromasts; anterior infra-orbital canal partially closed, with two pores and one free neuromast, but in juveniles and subadults all anterior infraorbital canal can be open; median portion of infra-orbital region with series of nine to eleven minute neuromasts; posterior infra-orbital canal closed, with two pores; preopercular canal closed in both dorsal and ventral portions with seven or eight pores; mandibular canal represented by two neuromasts, one in vertical through corner of mouth and the other anteriorly positioned in the lower jaw ventral portion. Longitudinal series of scales 27(3), 28(18), 29(9); transverse series of scales 7; circumpeduncular scales 10; predorsal scales 17(1), 18(18), 19(11).

##### Osteology.

**Figure 6.**
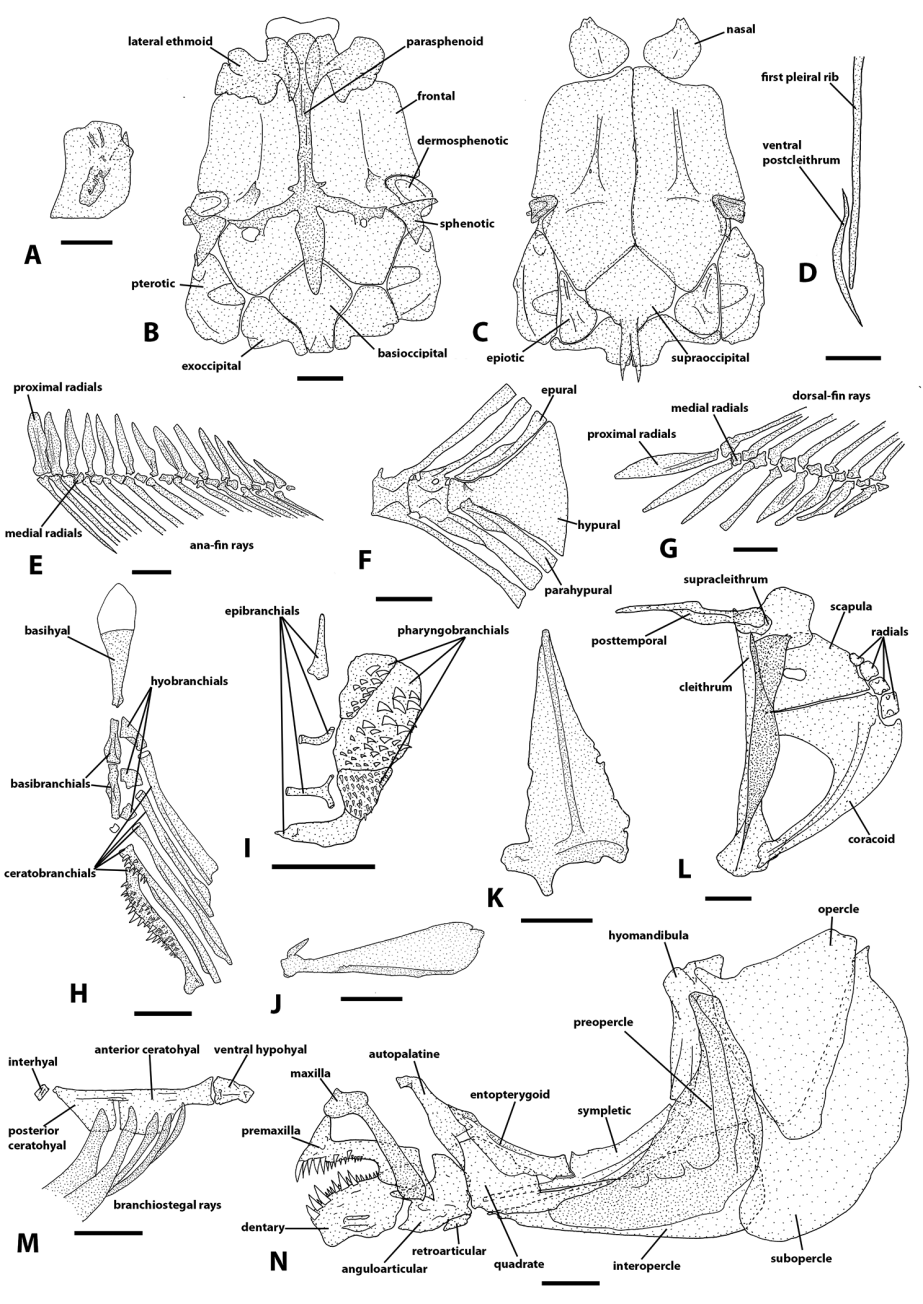
Osteological plate of “L.” *johnstoni* (SAIAB 35820) from the Upper Shire River, Mangochi, Malawi. **A** Lachrymal **B** neurocranium, ventral view **C** neurocranium dorsal view **D** ventral post-cleithrum and first pleural rib, lateral view **E** anal-fin radials and proximal radials, left lateral view **F** caudal-fin skeleton, left lateral view **G** dorsal-fin radials and proximal radials, left lateral view **H** left branchial arches ventral portion, ventral view **I** right dorsal portion of branchial arches, ventral view **J** urohyal, left lateral view **K** left basipterygium, dorsal view **L** left shoulder girdle, lateral view **M** right hyoid bar, lateral view **N** left jaws, jaws suspensorium and opercular apparatus, lateral view. Scale bars: 1 mm.

Osteological structures are presented in Fig. 6. Mesethmoid and vomer absent. Frontals, anterior margin, extending anteriorly between nasals. Parasphenoid medial process short, not contacting pterophenoid; anterior margin rounded. Lateral ethmoid overlapping with anterior portion of parasphenoid. Posterior process of supraoccipital long, reaching first vertebra. Lachrymal rectangular. Premaxillary and dentary teeth well developed. Retroarticular triangular or subtriangular. Dentary deep. Dorsal process of maxilla broad overlapping the ascending process of premaxilla; ventral process greatly reduced, consisting of a rounded ventromedial bulge. Ventral arm of maxilla broad, laterally expanded. Medial surface of premaxilla ascending process with a straight or slightly concave profile. Entopterygoid posterior portion and sympletic bone keel deep. Opercle triangular, anterodorsal process present. Anterior process of anterior ceratohyal does not extend ventrally to ventral hypohyal. Urohyal ventral margin concave. First and second basibranchials with expanded lateral bone flanges. Cartilaginous portion of basihyal shorter than osseous portion. Fourth ceratobranchial anterior third with teeth. Second pharyngobranchial plate with teeth. First epibranchial base, broad, more than three times anterior portion width. Supracleithrum rounded. Posttemporal rod-like, ventral arm absent. Cleithrum bony flange not covering scapula foramen. Ventral postcleithrum, slender, similar in width to adjacent first pelural rib. Basipterygium, posterior process, shorter or about the same size of medial process. Anal-fin proximal radials about the same length and parallel to each other. Hypurals completely fused. Parahypural proximal end overlapping the preural centrum. Total vertebrae 30(4) and 31(5), precaudal 13(2) and 14(7) and caudal 16(1), 17(4) and 18(4). First proximal radial of dorsal-fin between neural spine of vertebrae 16 and 17. First proximal radial of anal-fin between pleural rib of vertebrae 11 and 13. Gill rakers on first branchial arch 10(1) and 11(3). Branchiostegal rays 5.

##### Colouration in alcohol.

Overall colouration of body pale brown yellow with minute chromatophores sparsely distributed, and some organised chromatophores forming an inconspicuous reticulate pattern along flank scales margin (Fig. 3). Ventral surface scarcely pigmented. Pale brown chromatophores along whole mid-body line of flank. Head overall colouration yellowish brown. Brown chromatophores on dorsum of head. Minute chromatophores concentrated in the lower jaw and pre-orbital region, forming a distinct darker region. Iris silver, darker close to pupil; dark pigment concentrated on dorsal margin of eye. All fins hyaline in females, with melanophores sparsely concentrated only on fins membranes and along fin rays; melanophores forming small inconspicuous spots on male dorsal-fins, rarely forming two distinct bands; melanophores forming two distinct parallel dark bands in the anal-fin medial portion; region close to anal-fin rays insertion hyaline; caudal-fin with conspicuous dark blotches in the middle rays that could be organised in distinct bands or not; pectoral and pelvic-fin with melanophores sparsely concentrated on fins membranes and along fin rays. Female urogenital opening pocket scales with dark brown chromatophores.

##### Colouration in life.

**Males** (Fig. 4A). Flanks bright blue, with small scattered bright green dots. Cupric iridescent blotch on region just posterior to pectoral-fin. Dorsum yellow-brown. Ventral surface white between head and region anterior to pelvic-fin origin; bright blue between pelvic-fin and the end of the anal-fin insertion; ventral region of caudal peduncle whitish yellow. Side of head predominantly bluish silver, dorsal portion yellow-brown, post-orbital region with a distinct green bright blotch. Iris dark grey, light yellow close to pupil. Eye bright silver on dorsal portion. Lower jaw and pre-orbital region dark brown-grey, forming a distinct horizontal band. Pectoral-fin hyaline; all other fins yellowish brown with brown dots on fins arranged in distinct rows. Dorsal-fin with two to four rows; anal-fin with two to three rows; and caudal-fin with four to five rows, but in some specimens the brown dots are scattered over the fin and not arranged in distinct rows. Some males may present a distinct black distal margin on anal, dorsal and caudal-fins.

**Females** (Fig. 4B). Flanks bright blue. Cupric iridescent blotch on region just posterior to pectoral-fin. Dorsum yellow-brown. Venter white between head and region just anterior to urogenital opening; bright blue between region just anterior to urogenital opening and anal-fin insertion; whitish yellow along the anal-fin insertion to caudal peduncle. Scales around urogenital opening covered with dark chromatophores. Side of head predominantly bluish silver, ventral portion white, dorsal portion yellow-brown, post-orbital region with a distinct green bright blotch. Iris dark grey, light yellow close to pupil. Eye bright silver on dorsal portion. Lower jaw and pre-orbital region dark brown-grey, forming a distinct horizontal band. Pectoral and pelvic-fins hyaline; dorsal-fin orange, distal margin hyaline; anal-fin base hyaline, distal portion orange; caudal-fin orange, distal region hyaline.

##### Distribution and habitat.

“*Lacustricola*” *johnstoni* is a widespread species occurring in the Lower, Middle and Upper Zambezi River, including the Shire River and Lake Malawi, the Limpopo River, and is also present in the Okavango system (Fig. 2). The species is usually found associated with marginal vegetation along the banks of small and large rivers, or in swampy areas, as well as along the shores of Lake Malawi and Lake Kariba.

#### Lacustricola myaposae

Taxon classificationAnimaliaCyprinodontiformesPoeciliidae

(Boulenger, 1908)

BDCD42AF-C1CE-50BD-A29E-A1DF0C4DA108

Figures 7 , 8

Haplochilus myaposae Boulenger 1908:232 [original description: Myaposa River, Zululand, KwaZulu-Natal, South Africa].

##### Material examined.

BMNH 1907.4.17.88, Lectotype; Myaposa River, Kwazulu-Natal, South Africa. Examined by photographs and x-rays. SAIAB 96619, 10 (2 C&S), 18.1–27.9 mm SL; Mhlathuze, KwaZulu-Natal, South Africa 28°50'18"S, 31°54'41"E; col: B. Ellender, O. Weyl & R. Karsing; 27 May. 2010. – SAIAB 88658, 31 (6 C&S), 21.3–38.0 mm SL; Bridge at Mseleni, KwaZulu-Natal, South Africa, 27°21'49"S, 32°31'33"E; col: B. Kramer, E. Swartz, P.T. Maake; 31 Oct. 2009. – SAIAB 96560, 1, 30.2 mm SL; Nseleni River Nature Reserve, KwaZulu-Natal, South Africa, 28°41'57"S, 32°0'4"E; col: R. Jones, O. Weyl; B. Ellender & R. Karsing; 23 May. 2010. – SAIAB 86637, 1, 34.0 mm SL; St Lucia area 2, KwaZulu-Natal, South Africa, 28°20'44"S, 32°21'14"E; col: R. Karssing, J. Craigie, S. Khubela, R. Ndlhovu, A. Xoswa; 08 Sep. 2009. – SAIAB 47128, 1, 40.5 mm SL; KwaZulu-Natal, South Africa; 31 Jan. 1989. – SAIAB 83143, 2, 38.1–38.2 mm SL; Greater St Lucia Wetland Park, Ozabeni, Ovalweni crossing, KwaZulu-Natal, South Africa, 27°38'59"S, 32°38'9"E; col: J.D. Craigie & R. Karssing; 29 May. 2007. – SAIAB 96591, 9 (2 C&S), 21.1–36.2 mm SL; Upper Nseleni in sugar Estate, Richards Bay, KwaZulu-Natal, South Africa, 28°40'27"S, 31°57'51"E; col: B. Ellender, O. Weyl & R. Karsing; 24 May. 2010. – SAIAB 83149, 3, 34.7–38.1 mm SL; Greater St Lucia Wetland Park, Ozabeni, Samango crossing, KwaZulu-Natal, South Africa, 27°37'3"S, 32°33'2"E; col: N. Rivers-Moore & R. Karssing; 30 May. 2007. – SAIAB 208915, 6; Makat Farm in Jamela, Mposa River, a tributary of the Nseleni River, KwaZulu-Natal, South Africa, 28°39'18"S, 32°01'48"E; col: A. Chakona, N. Mazungula & B. Motshegoa; 4 Sep. 2015.

##### Diagnosis.

“*Lacustricola*” *myaposae* is distinguished from all congeners from the “L.” katangae clade by the absence of a zigzag black mark along the flank (vs. presence); and from congeners belonging to the “L.” hutereaui clade by the absence of barred dorsal, anal and caudal-fins and also by the absence of a conspicuous reticulate pattern on scales margin. It is further distinguished from all congeners except “L.” *johnstoni* and “L.” *moeruensis* by the presence of an orange dorsal, anal and caudal-fins in females (vs. hyaline); it is distinguished from “L.” *johnstoni* and “L.” *moeruensis* by the presence of light purple colouration in the posterior region of flank (vs. absence); an orange pectoral-fin in males (vs. hyaline); and by a distinct colouration pattern in both dorsal and anal-fins in which melanophores become continuously more concentrated close to fin margins, forming a grey zone before the margin become entirely dark (vs. absence of this colouration pattern). “*Lacustricola*” *myaposae* is further distinguished from “L.” *johnstoni* by a deeper body profile, males body depth 26.0–30.9% of SL (vs. 20.6–24.4%of SL), females body depth 22.8–25.1% of SL (vs. 19.7–22.5% of SL); a longer dorsal-fin base length in males 11.9–13.1%of SL (vs. 8.7–11.6% of SL) and in females 10.8–11.6% SL (vs. 7.3–10.1% SL); and a deeper head in males 70.7–79.8% of HL (vs. 61.9–67.0% of HL) and in females 66.6–69.6% of HL (vs. 59.4–63.5% of HL). Other morphometric characters presenting a slight overlap but useful in distinguishing “L.” *myaposae* from “L.” *johnstoni* are: a deeper caudal peduncle, 14.0–17.1% of SL in males and 12.9–13.7% of SL in females (vs. 12.6–14.5% of SL in males and 11.2–12.7% of SL in females); a shorter caudal-fin, 27.5–30.2% of SL in males and 25.3–28.5% of SL in females (vs. 30.1–33.8% of SL in males and 28.9–31.2% of SL in females); and a deeper head, 63.1–67.9% of HL in males and 65.3–68.1% of HL in females (vs. 57.1–63.2% of HL in males and 59.0–64.7% of HL in females).

##### Description.

Morphometric data are presented in Table 1. Maximum recorded adult size 39.0 mm SL. Dorsal profile of body approximately straight to slightly convex from snout tip to dorsal-fin origin; convex along dorsal-fin base, and nearly straight on caudal peduncle. Ventral profile convex from lower jaw to beginning of anal-fin base; slightly convex along the anal-fin base and nearly straight on caudal peduncle. Caudal peduncle slightly deeper in males. Anterior portion of body laterally compressed, becoming more compressed behind anal-fin origin.

**Figure 7.**
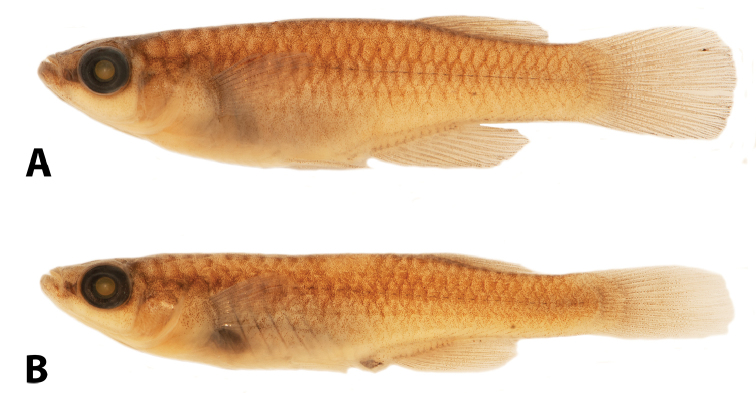
“*Lacustricola*” *myaposae* preserved colouration: **A** male SAIAB 96591, 31.7 mm SL**B** female SAIAB 96591, 35.7 mm SL; from Nseleni River, KwaZulu-Natal, Republic of South Africa.

Dorsal-fin rounded in males not reaching caudal-fin base; its origin in vertical between 6^th^ and 7^th^ anal-fin rays. (Fig. 7) Anal-fin rounded in males, tip not reaching vertical through dorsal-fin tip. Dorsal and anal-fins are not elongated in females. Caudal-fin slender. Pectoral-fin elliptical, its posterior margin reaching vertical just behind pelvic-fin base. Pelvic-fin length in males longer than in females, reaching urogenital papillae aperture; short in females, tip reaching region just before urogenital opening. Pelvic-fin bases medially separated by interspace broader than width of each pelvic-fin base. Dorsal-fin rays 8(7), 9(20), 10(10) and 11(2); anal-fin rays 12(1), 13(9), 14(14) and 15(15); caudal-fin rays 20(3), 21(7), 22(9), 23(6), 24(9), 25(3) and 26(1); pectoral-fin rays 11 (2), 12(18) and 13(19); pelvic-fin rays (6).

Frontal squamation G-patterned (Fig. 5). Head neuromasts placed in shallow grooves. Cephalic lateral line system: anterior portion of supraorbital sensory canal open, with three neuromasts, anteriormost one anteriorly displaced from the other two; posterior portion open, with three exposed neuromasts; anterior infra-orbital canal partially closed, with three pores and one free neuromast, but in juveniles and subadults all anterior infraorbital canal can be opened with three exposed neuromasts; median portion of infra-orbital region with series of eight to nine minute neuromasts; posterior infra-orbital canal closed, with two pores; preopercular canal closed in both dorsal and ventral portions with seven pores; mandibular canal represented by two neuromasts, one in vertical through corner of mouth and the other anteriorly positioned in the lower jaw ventral portion. Longitudinal series of scales 26(14), 27(11), 28(8), 29(1); transverse series of scales (7); circumpeduncular scales (10); predorsal scales 16(17), 17(16), 18(3).

##### Osteology.

Osteological structures are presented in Fig. 6. Mesethmoid and vomer absent. Frontals, anterior margin, extending anteriorly between nasals. Parasphenoid medial process short, not contacting pterosphenoid; anterior margin rounded. Lateral ethmoid overlapping with anterior portion of parasphenoid. Posterior process of supraoccipital long, reaching first vertebra. Lachrymal rectangular. Premaxillary and dentary teeth well developed. Retroarticular triangular or subtriangular. Dentary deep. Dorsal process of maxilla broad overlapping the ascending process of premaxilla; ventral process greatly reduced, consisting of a rounded ventromedial bulge. Ventral arm of maxilla broad, laterally expanded. Medial surface of premaxilla ascending process with a straight or slightly concave profile. Entopterygoid posterior portion and sympletic bone keel deep. Opercle triangular, anterodorsal process present. Anterior process of anterior ceratohyal does not extend ventraly to ventral hypohyal. Urohyal ventral margin concave. First and second basibranchials with expanded lateral bone flanges. Cartilaginous portion of basihyal shorter than osseous portion. Fourth ceratobranchial anterior third with teeth. Second pharyngobranchial plate with teeth. First epibranchial base, broad, more than three times anterior portion width. Supracleithrum rounded. Posttemporal rod-like, ventral arm absent. Cleithrum bony flange not covering scapula foramen. Ventral postcleithrum slender, similar in width to adjacent first pelural rib. Basipterygium, posterior process, shorter or about the same size of medial process. Anal-fin proximal radials about the same length and parallel to each other. Hypurals completely fused. Parahypural proximal end overlapping the preural centrum. Total vertebrae 28(1), 29(8) and 30(1), precaudal 12(9) and 13(1) and caudal 16(2), 17(7) and 18(1). First proximal radial of dorsal-fin between neural spine of vertebrae 13 and 14. First proximal radial of anal-fin between pleural rib of vertebrae 11 and 12. Gill rakers on first branchial arch 9(1), 10(2) and 11(1). Branchiostegal rays 5.

##### Colouration in alcohol.

Overall colouration of body pale brownish yellow with minute chromatophores sparsely distributed, and some organised chromatophores forming an inconspicuous reticulate pattern along margins of flank scales, more conspicuous along the longitudinal series of scales on mid-body line of flank (Fig. 7). Ventral surface scarcely pigmented. Pale brown chromatophores along whole mid-body line of flank. Head overall colouration yellowish brown. Dark brown chromatophores on dorsum of head. Minute chromatophores concentrate in the lower jaw and pre-orbital region, forming a distinct darker region. Iris silver, darker close to pupil; dark pigment concentrated on dorsal margin of eye. All fins hyaline in females, with melanophores sparsely concentrated only on fins membranes and along fin rays; high concentration of melanophores on males dorsal-fin, with small hyaline spots on fin membrane, close to rays insertion; dorsal-fin margin dark; melanophores on anal-fin becoming continuously more concentrated close to fin margin; region close to anal-fin rays insertion hyaline; caudal-fin with conspicuous dark blotches in the middle rays that could be organised in distinct bands or not, melanophores on fin distal portion becoming continuously more concentrate close to fin margin; pelvic-fin dark, with numerous small chromatophores; pectoral-fin with chromatophores sparsely concentrated on fins membranes and along fin rays. Female urogenital opening pocket scales with few sparse dark brown chromatophores.

##### Colouration in life.

**Figure 8.**
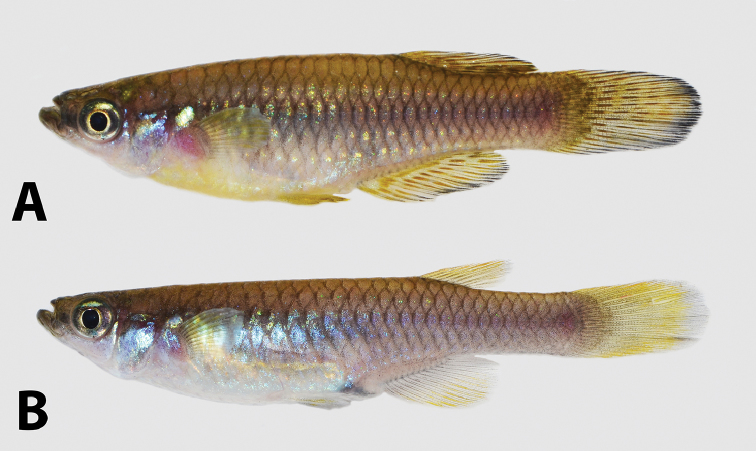
“*Lacustricola*” *myaposae* colouration in life: **A** male SAIAB 208915, 33.0 mm SL**B** female SAIAB 208915, 35.2 mm SL; from Nseleni River, KwaZulu-Natal, Republic of South Africa.

**Males** (Fig. 8A). Flanks yellow brown, scattered with yellow-green metallic dots along the flank; posterior region of flank purple, more conspicuous posteriorly on caudal peduncle. Small cupric iridescent blotch on flank region just posterior to pectoral-fin. Dorsum yellow-brown. Ventral surface white between lower jaw and opercle margin; light yellow between opercle margin and urogenital opening; greyish brown between urogenital opening and caudal peduncle. Side of head predominantly bluish silver, dorsal portion yellow-brown, post-orbital region with a distinct green bright blotch. Iris dark grey, light yellow close to pupil. Eye bright silver on dorsal portion. Lower jaw and pre-orbital region dark brown-grey, forming a distinct horizontal band. Pectoral-fin base hyaline, distal portion orange; pelvic-fin yellow with dark chromatophores on fins tip; anal-fin with two orange-brown rows and melanophores on becoming continuously more concentrate close to fin margin; dorsal-fin orange-yellow with two-three dark bars on its posterior portion, margin black; caudal-fin orange-yellow with three vertical bands that could be organised in distinct bands or not, melanophores on fin distal portion becoming continuously more concentrate close to fin margin.

**Females** (Fig. 8B). Flanks bright blue, scattered with green metallic dots along the flank; posterior region of flank purple, more conspicuous posteriorly on caudal peduncle. Cupric iridescent blotch on region just posterior to pectoral-fin. Dorsum yellow-brown. Venter white between head and pelvic-fin insertion; bright blue between pelvic-fin and end of caudal-fin; grey on caudal peduncle. Scales around urogenital opening covered with dark chromatophores. Side of head predominantly bluish silver, ventral portion white, dorsal portion yellow-brown, post-orbital region with a distinct green bright blotch. Iris dark grey, light yellow close to pupil. Eye bright silver on dorsal portion. Lower jaw and pre-orbital region dark brown-grey, forming a distinct horizontal band. Pectoral-fin first rays orange-brown; pelvic-fin hyaline, with a faint orange colouration on fin base; dorsal-fin orange, distal margin hyaline; anal-fin base hyaline, distal portion orange; caudal-fin orange, distal region hyaline.

##### Distribution and habitat.

“*Lacustricola*” *myaposae* is only known from the coastal river drainages and lacustrine systems in Kwazulu-Natal Province of South Africa to coastal lagoons south of the Maputo River in Mozambique (Fig. 2). The species is usually found associated with marginal vegetation along the banks of small and large rivers in freshwater. Despite occurring in a coastal area, little is known about the salinity tolerance capacity of “L.” *myaposae*.

##### Remarks.

Only one specimen was found among the syntypes (original catalogue number BMNH 1907.4.17.88-89) of “*Lacustricola*” *myaposae*, one specimen is missing (pers. comm. James Maclaine), thus this remaining individual was designated as the Lectotype (BMNH 1907.4.17.88).

## Discussion

The present study provided the first insight into the phylogenetic diversity and relationships between the southern Africa “*Lacustricola*” species. Among the major challenges and impediments for taxonomic studies within this genus is the assumption that some species (e.g. “L.” katangae, “L.” *johnstoni*, and “L.” hutereaui) are broadly distributed over southern Africa and the lack of detailed information about species boundaries. Thus, this paper is a first attempt to approach both impediments, through a combined broad COI mitochondrial gene sampling of most southern Africa “*Lacustricola*” species and the redescriptions of “L.” *myaposae* and “L.” *johnstoni*, the latter a species that has been considered to be widespread in southern Africa. However, it is worth mentioning that despite no evidence of introgression within the Procatopodidae, considering the presence of sympatric species this is a possibility, and phylogenetic relationships based only on mitochondrial DNA may fail in identifying that, and not necessarily reflect the species phylogeny. In addition, another main concern is that given the limited COI sampling for some regions, the results must be seen as a first effort in investigating the little-known southern Africa “*Lacustricola*”.

Both, ML and BI analyses supported “L.” hutereaui and “L.” katangae as belonging to distinct groups, each one with clear distinct colouration patterns. The “*Lacustricola*” katangae group (including also “L.” mediolateralis) is easily recognised by the presence of a zigzag pattern black band along the flank, whereas specimens belonging to the “L.” hutereaui group have barred dorsal, anal and caudal-fins and a conspicuous reticulate pattern on scale margins. Despite generally having broadly similar distribution ranges, the contrasting genetic patterns between the “L.” katangae and “L.” hutereaui groups suggest that these groups had different evolutionary histories in response to the paleogeographic and paleoclimatic events in the region. “*Lacustricola*” katangae was found to be a single widely distributed species with small genetic divergence among haplotypes and no discernible pattern of geographic structuring across its range which extends from the KwaZulu Natal Province of South Africa in the south to the Congo system. In contrast, the “L.” hutereaui group contained two distinct lineages, the first comprising samples from the Okavango and Zambezi systems, and another one represented by a single haplotype from the Lualaba River in the Congo system. There are a number of possibilities regarding taxonomic status of the two lineages identified within the “L.” hutereaui group. Firstly, they could potentially represent two species that are new to science. Secondly, one of them could represent “L.” hutereaui from the northern savannahs of the Congo in the Democratic Republic of Congo, or they could also potentially represent two known synonyms of “L.” hutereaui, namely “L.” *baudoni* (Myers, 1924b) from northern Congo savannahs in Central African Republic, and “L.” *chobensis* (Fowler, 1935) from the Chobe River at Kasane, close to the Zambezi river confluence. Determination of the taxonomic status of these lineages and evaluation of the validity of the two junior synonyms of “L.” hutereaui will require inclusion of topotypic samples from the type localities of “L.” hutereaui and “L.” *baudoni* which were not available for the present study.

One of the key contributions of this paper was provision of detailed redescriptions of “L.” *johnstoni* and “L.” *myaposae*. The lack of a clear diagnosis for “L.” *johnstoni* in particular, resulted in this species essentially becoming a “waste basket taxon” for all slender-bodied topminnows with bluish colouration. This was demonstrated by the fact that several samples from a number of ichthyological collections that were labelled as “L.” *johnstoni* did not form a distinct clade, but instead, some of these samples clustered with other species, for example “L.” macrurus, “L.” centralis and “L.” *myaposae*. The incorporation of COI sequences from the type locality of “L.” *johnstoni* in the Shire River allowed identification of “L.” *johnstoni* s. s. and revealed, for the first time, that specimens with a slender body and bluish colouration are not necessarily conspecific with this species nor are they closely related to it. For example, Bragança and Costa (2019) erroneously classified sample (UFRJ 10873) as “L.” *johnstoni*, which in this study has been recovered as “L. ” aff. johnstoni ‘Okavango’, while a juvenile specimen that was tentatively identified as “L.” *matthesi* (UFRJ 10894) by the same authors has been found to correspond to “L.” *johnstoni* in the present study. Two new candidate species were recognised, “L. ” aff. johnstoni ‘Congo’ from the upper Lualaba River in the Congo system that is sister to “L.” macrurus, a species known from the Kwanza River and upper Kasai River, a Congo River tributary, and “L. ” aff. johnstoni ‘Okavango’ from the Okavango and Upper Zambezi drainages that is sister to “L.” centralis, the only known southern Africa *Lacustricola* species occurring in the eastern African Malagarasi River. The distinction between “L.” *johnstoni* and “L. ” aff. johnstoni ‘Okavango’ in the field or relying only on morphology could be really challenging because both have the same body profile and occur sympatrically in the Okavango Delta. Thus, we consider the COI barcoding approach a useful tool in distinguishing both species, if their distinctiveness is confirmed with additional data apart from COI-barcoding.

Following an integrative taxonomy perspective, in addition to the COI molecular haplotype analysis, detailed information on the morphology, colouration pattern and osteology were presented for the first time for the until then little known “L.” *johnstoni* and “L.” *myaposae*. A detailed redescription of “L.” *johnstoni*, based on specimens from and close its type locality is herein considered the first step before describing new species and investigating more deeply the genetic and species diversity within the broadly distributed “L.” *johnstoni*. Despite the broad sampling, further studies directed to fill important gaps, applying different species delimitation methods and maybe incorporating different markers are needed to better understand the diversity within “L.” *johnstoni* and other southern Africa *Lacustricola* groups.

## Supplementary Material

XML Treatment for Lacustricola johnstoni

XML Treatment for Lacustricola myaposae

## Supplementary materials

Supplementary material 1 Species localities and Genbank Acession numbers: acession numbers in bold refers to sequences developed in the present study Data type species data https://binary.pensoft.net/file/396606This dataset is made available under the Open Database License (http://opendatacommons.org/licenses/odbl/1.0/). The Open Database License (ODbL) is a license agreement intended to allow users to freely share, modify, and use this Dataset while maintaining this same freedom for others, provided that the original source and author(s) are credited.Pedro H.N. Bragança, Ryan M. van Zeeventer, Roger Bills, Denis Tweddle, Albert Chakona

## References

[B1] Ahl E (1928) Beiträge zur Systematik der africanischen Zahnkarpfen. Zoologischer Anzeiger 79: 115–116.

[B2] Bragança PHN Costa WJEM (2018) Time-calibrated molecular phylogeny reveals a Miocene–Pliocene diversification in the Amazon miniature killifish genus *Fluviphylax* (Cyprinodontiformes: Cyprinodontoidei). Organisms, Diversity and Evolution 18: 345–353. 10.1007/s13127-018-0373-7

[B3] Bragança PHN Costa WJEM (2019) Multigene fossil-calibrated analysis of the African lampeyes (Cyprinodontoidei: Procatopodidae) reveals an early Oligocene origin and Neogene diversification driven by palaeogeographic and palaeoclimatic events. Organisms, Diversity and Evolution 19: 1–18. 10.1007/s13127-019-00396-1

[B4] Bragança PHN Amorim PF Costa WJEM (2018) Pantanodontidae (Teleostei, Cyprinodontiformes), the sister group to all other cyprinodontoid killifishes as inferred by molecular data. Zoosystematics and Evolution 94: 137–145. 10.3897/zse.94.22173

[B5] Boulenger GA (1906) Fourth contribution to the ichthyology of Lake Tanganyika. Report on the collection of fishes made by Dr. W. A. Cunnington during the Third Tanganyika Expedition, 1904–1905. Transactions of the Zoological Society of London 17: 537–601. 10.1111/j.1096-3642.1905.tb00037.x

[B6] Boulenger GA (1908) On a collection of freshwater fishes, Batrachians and Reptiles from Natal and Zululand, with descriptions of new species. Annals of the Natal government. 1: 219–239.

[B7] Boulenger GA (1912) Description d’un poisson nouveau du genre *Haplochilus* provenent du Katanga. Revue de Zoologie Africaine 2: 47–48.

[B8] Boulenger GA (1913) Sur une petite collection de poissons récueillis das l’Uelé, par la mission dirigée par M. Hutereau. Revue de Zoologie Africaine 2(2): 155–161.

[B9] Chakrabarty P (2010) Genetypes: a concept to help integrate molecular phylogenetics and taxonomy. Zootaxa 2632: 67–68. 10.11646/zootaxa.2632.1.4

[B10] Clausen HS (1967) Tropical Old World cyprinodonts. Akademisk Forlag, Copenhagen, 64 pp.

[B11] Costa WJEM (1988) Sistemática e distribuição do complexo de espécies *Cynolebias minimus* (Cyprinodontiformes, Rivulidae), com a descrição de duas espécies novas. Revista Brasileira de Zoologia 5: 557–570. 10.1590/S0101-81751988000400004

[B12] Costa WJEM (1996) Relationships, monophyly and three new species of the neotropical miniature poeciliid genus *Fluviphylax* (Cyprinodontiformes: Cyprinodontoidei). Ichthyological Exploration of Freshwaters 7: 111–130.

[B13] Costa WJEM (2006) Descriptive morphology and phylogenetic relationships among species of the Neotropical annual killifish genera *Nematolebias* and *Simpsonichthys* (Cyprinodontiformes: Aplocheiloidei: Rivulidae). Neotropical Ichthyology 4: 1–26. 10.1590/S1679-62252006000100001

[B14] Darriba D Taboada GL Doallo R Posada D (2012) jModelTest 2: more models, new heuristics and parallel computing. Nature Methods 9: 772. 10.1038/nmeth.2109 PMC4594756 22847109

[B15] Felsenstein J (1985) Confidence limits on phylogenies: an approach using the bootstrap. Evolution 39: 783–791. 10.1111/j.1558-5646.1985.tb00420.x 28561359

[B16] Folmer O Black M Hoeh W Lutz R Vrijenhoek R (1994) DNA primers for amplification of mitochondrial cytochrome c oxidase subunit I from diverse metazoan invertebrates. Molecular Marine Biology and Biotechnology 3: 294–299. 7881515

[B17] Fowler HW (1935) Scientific results of the Vernay-Lang Kalahari expedition, March to September 1930. Fresh-water fishes. Annals of the Transvaal Museum 16: 251–293.

[B18] Ghedotti MJ (2000) Phylogenetic analysis and taxonomy of the poecilioid fishes (Teleostei: Cyprinodontiformes). Zoological Journal of the Linnean Society 130: 1–53. 10.1111/j.1096-3642.2000.tb02194.x

[B19] Gosline WA (1949) The sensory canals of the head in some cyprinodont fishes, with particular reference to the genus *Fundulus*. Occasional Papers of the Museum of Zoology from University of Michigan 519: 1–17.

[B20] Günther A (1894) Second report on the reptiles, batrachians and fishes transmitted by Mr. H.H. Johnston C.B. from British Central Africa. Proceedings of the Zoological Society of London 1894: 616−628.

[B21] Helmstetter AJ Papadopulos AST Igea J Van Dooren TJM Leroi AM Savolainen V (2016) Viviparity stimulates diversification in an order of fish. Nature Communications 7: 11271. 10.1038/ncomms11271 PMC4832061 27070759

[B22] Hoedeman JJ (1956) Die bisher beschriebenen Formen und Arten der Gattung *Rivulus* Poey. Aquarium Terrarium 1956: 199–202.

[B23] Huber JH (1999) Updates to the phylogeny and systematics of the African lampeye schooling cyprinodonts (Cyprinodontiformes: Aplocheilichthyinae). Cybium 23: 53–77.

[B24] Kumar S Stecher G Tamura K (2016) MEGA7: Molecular evolutionary genetics analysis version 7.0 for bigger datasets. Molecular Biology and Evolution 33: 1870–1874. 10.1093/molbev/msw054 27004904 PMC8210823

[B25] Myers GS (1924) New genera of African poeciliid fishes. Copeia 129: 42–43. 10.2307/1436003

[B26] Myers GS (1924b) A new Poeciliid fish of the genus *Micropanchax* from Ubangui. American Museum Novitates 122: 1−3.

[B27] Parenti LR (1981) A phylogenetic and biogeographic analysis of cyprinodontiform fishes (Teleostei, Atherinomorpha). Bulletin of the American Museum of Natural History 168: 335–357.

[B28] Pohl M Milvertz FC Meyer A Vences M (2015) Multigene phylogeny of cyprinodontiform fishes suggests continental radiations and a rogue taxon position of *Pantanodon*. Vertebrate Zoology 65: 37–44.

[B29] Poll M (1967) Contribution à la faune ichthyologique de l’Angola. Publicações Culturais, Companhia de Diamantes de Angola (DIAMANG), Lisbon, 381 pp.

[B30] Poll M Lambert JG (1965) Contribution a L’etude systematic et zoogeographique des Procatopodinae de L’Afrique central (Pisces, Cyprinodontidae). Bulletin des Séances. Académie Royale des Sciences d’Outre-Mer 2: 615–631.

[B31] Pollux BJA Meredith RW Springer MS Garland T Reznick DN (2014) The evolution of the placenta drives a shift in sexual selection in live-bearing fish. Nature 513: 233–236. 10.1038/nature13451 25043015

[B32] Reznick DN Furness AI Meredith RW Springer MS (2017) The origin and biogeographic diversification of fishes in the family Poeciliidae PLoS One 12: e0172546. 10.1371/journal.pone.0172546 PMC5344339 28278162

[B33] Ronquist F Teslenko M Van der Mark P Ayres D Darling A Hohna Set al. (2012) MrBayes 3.2: Efficient Bayesian phylogenetic inference and model choice across a large model space. Systematic Biology 61: 539–542. 22357727 10.1093/sysbio/sys029 PMC3329765

[B34] Seegers L (1996) The Fishes of the Lake Rukwa Drainage. Annales du Musée Royal de l’Afrique Centrale, Sciences Zoologiques 287: 1–407.

[B35] Skelton PH (2001) A Complete Guide to the Freshwater Fishes of Southern Africa. Struik Publishers, Cape Town, 395 pp.

[B36] Sunnucks P England PR Taylor AC Hales DF (1996) Microsatellite and chromosome evolution of parthenogenetic Sitobion aphids in Australia. Genetics 144: 747–756. 8889535 10.1093/genetics/144.2.747 PMC1207565

[B37] Taylor WR Dyke V (1985) Revised procedures for staining and clearing small fishes and other vertebrates for bone and cartilage study. Cybium 9: 107–109.

[B38] Van der Zee JR Sonnnenberg R Munene JJMM (2015) *Hypsopanchax stiassnyae*, a new poeciliid fish from the Lulua River (Democratic Republic of Congo) (Teleostei: Cyprinodontiformes). Ichthyological Exploration of Freshwaters 26: 87–96.

[B39] Wildekamp RH Romand R Scheel JJ (1986) Cyprinodontidae. In: Daget J Gosse JP Van den Audenaerde T (Eds) Check−list of the freshwater fishes of Africa 2 (CLOFFA 2).ISNB, MRAC, ORSTOM, Brussels, Tervuren, Paris, 165–276.

[B40] Zwickl DJ (2006) Genetic algorithm approaches for the phylogenetic analysis of large biological sequence datasets under the maximum likelihood criterion. PhD thesis, Austin, Texas, United States of America: University of Texas at Austin.

